# Nasopharyngeal, tongue and laryngeal cancer in Southern Ethiopia: a seven-year retrospective cross-sectional review

**DOI:** 10.3332/ecancer.2024.1784

**Published:** 2024-10-08

**Authors:** Achamyelesh Gebretsadik, Netsanet Bogale, Dereje Geleta, Nebiyu Melaku, Dubale Dulla

**Affiliations:** 1 School of Public Health, College of Medicine and Health Sciences, Hawassa University, 1560 Hawassa, Ethiopia; 2 Faculty of Medicine, College of Medicine and Health Sciences, Hawassa University, 1560 Hawassa, Ethiopia; 3 Maternal and Child Health Core Process, Southern Nation Nationalities and People Regional Health Bureau, 1560 Hawassa, Ethiopia; 4 Department of Midwifery, College of Medicine and Health Sciences, Hawassa University, 1560 Hawassa, Ethiopia; a https://orcid.org/0000-0002-0060-2103; b https://orcid.org/0000-0003-0202-763X

**Keywords:** nasopharyngeal, tongue and laryngeal cancer, epidemiology, Southern Ethiopia

## Abstract

**Background:**

The burden of cancer is increasing globally and is having a negative impact on people’s physical, mental and financial health. On the other hand, developing countries are not progressing to prevent the disease at the same rate as the disease burden increases. The development of strategies for cancer prevention, control and treatment that contribute to the community’s improved health requires knowledge of cancer epidemiologic data. There is relatively little epidemiologic evidence of nasopharyngeal, tongue and laryngeal cancer in southern Ethiopia. This study aimed to assess the epidemiological burden of nasopharyngeal, tongue and laryngeal cancer among patients treated at Hawassa University Comprehensive and Specialised Hospital (HUCSH) between 2013 and 2019.

**Methods:**

A cross-sectional retrospective review was conducted among 3,002 patients who attended the oncologic care at HUCSH. Data were retrieved between February and May 2020. Data were entered using Epi-data version 3.1 and the data were then exported to IBM SPSS version 22 (IBM Corporation, Armonk, NY, USA) for further processing and analysis. A descriptive analysis was done.

**Result:**

A total of 280 (9.3%) new head and neck cancer (HNC) patients were identified over a period of 7 years. Nasopharyngeal cancer accounts for more than one-fourth (26.4%) of all HNCs, followed by tongue 15% and laryngeal 14.6% cancers. Males constituted nearly two-thirds of the cases. The overall caseloads doubled over the retrieved years.

**Conclusion:**

According to this study, nasopharyngeal, tongue and laryngeal cancer is a more prominent cause of morbidity. According to place, person and time, the frequency of nasopharyngeal, tongue and laryngeal cancer steadily rose in both sexes and across all age categories. Therefore, immediate intervention is needed nationwide to monitor the disease’s explosive growth.

## Background

Cancer has a significant financial impact on society in both economically developed and poor nations; the least resource-rich nations still carry the bulk of the load. Population increase and aging, the rising incidence of established risk factors, and changing reproductive patterns related to urbanisation and economic growth have an impact on the likelihood of developing cancer [1, 2]. Between 2012 and 2018, studies on cancer incidence and mortality showed that there were roughly 8.2 to 9.6 million cancer-related deaths and 11 to 18.7 million new cancer cases worldwide [1, 3–5].

Globally and in Sub-Saharan Africa, which includes Ethiopia, head and neck cancers (HNCs) are ranked sixth and third, respectively [6, 7]. The prevalence of head and neck malignancies has increased in the United States of America; notwithstanding some fluctuations [6–8]. Pharyngeal cancer was found to be the most common cancer in most studies of HNCs conducted around the world [9–11], besides laryngeal cancer was shown to be the most common cancer in other studies [12–15]. In several studies, also oral cancers were the most commonly reported cancers [16–23].

However, staging of HNC based on each anatomical site, the American Joint Committee on Cancer (AJCC) system generally classify as early stages (I and II) and later stages (III and IV), according to a study conducted in the Netherlands, the incidence of oral, oro-pharyngeal and hypo-pharyngeal carcinoma has increased significantly over time [24–26]. Oral and oropharyngeal cancers accounted for the highest grade of HNC, according to a study conducted by Addis Ababa University [27]. The exact burden of HNCs in Africa is unknown due to a variety of factors, including poor case recording, unclear nomenclature and a lack of functional cancer registries [6, 28, 29]. All of these factors could be linked to similar challenges, such as a lack of human, technical and financial resources [19, 25].

The difficulties and complications of treating HNC in low-resource environments have been brought to light by more recent research. Early detection and community awareness are crucial for enhancing the prognosis of Ethiopian HNC patients. The researchers reported that late-stage presentation continues to be a major obstacle to receiving an appropriate course of therapy. This is frequently because of poor public health education regarding cancer symptoms and restricted access to specialised healthcare services. Furthermore, noted that there was an urgent need for improved training for healthcare professionals in the area, and they suggested that putting an emphasis on capacity-building could increase treatment efficacy and diagnostic accuracy. These results highlight the significance of addressing HNC from multiple angles, combining systemic changes in healthcare infrastructure and education with medical developments [26, 27].

This study intends to close the knowledge gaps about the epidemiology of tongue, laryngeal and nasopharyngeal cancers (NPCs) in the southern parts of Ethiopia, particularly at Hawassa University Comprehensive and Specialised Hospital (HUCSH). By doing this, it intends to provide vital information that can guide the distribution of resources and healthcare policies, ultimately enhancing the quality of cancer care and outcomes in the area. Additionally, knowing the local epidemiology of these malignancies can aid in the creation of focused prevention and treatment plans that are adapted to the particular environmental and demographic characteristics of the region. This is especially crucial considering the high incidence of head and neck malignancies worldwide and the particular difficulties in treating these illnesses that low-resource environments experience [28, 29].

## Methods

### Study setting and period

This study is part of a larger project conducted on the economic burden of cancers in HUCSH, southern Ethiopia, and the method used to conduct it was published in the Journal of Cancer Control in 2021 [30]. The HUCSH in Hawassa is where the study was conducted. The only cancer treatment and diagnosis facility for residents of southern Ethiopia, encompassing Sidama, Southern Nation Nationalities People (SNNP), Oromia, and other nearby regions, is this government-owned hospital. The hospital today provides a variety of treatment options, including surgery, chemotherapy and hormonal treatments, for more than 18 million patients.

### Data collection, management and analysis

A cross-sectional study design was used. Data were extracted from the patient’s card and registration book retrospectively using a structured checklist. All data between 2013 and 2019 were included. Records of all patients with a cancer diagnosis in registration books were retrieved for examination by obtaining the card number of each patient registered in an oncology unit throughout the study period. Then, HNCs were searched for in all cancer patient cards. The research team followed a standard checklist to gather data from February through May 2020. Data were entered using Epi-data version 3.1 and then exported for further processing and analysis to IBM SPSS version 22 (IBM Corporation, Armonk, NY, USA). AJCC, eighth edition was used for the Classification of cancer stages. Both descriptive and trend analyses were carried out.

## Result

### Epidemiologic distributions of cancer

Over the course of seven years, 280 HNC cases with ages ranging from 10 to 90 were extracted from the full records of all new cancer cases. Of these, 26.4% are NPCs, with tongue and laryngeal cancers accounting for 15% and 14.6% of all HNCs, respectively. A few others were accounted for by the gingival, lip, mandibular, oral cavity, paranasal sinus, submandibular, soft palate and paraganglioma Table 1.

The mean age for these patients was 41.9 years. Nearly 44% of all nasopharyngeal, tongue and laryngeal cancer patients were married. The majority of the cases came from Oromia region 121 (43.2%) and SNNPR 157(56.1%). While laryngeal and NPCs were frequently seen in the Oromia region, conjunctiva and tongue cancers were common in SNNPR (Table 2).

The majority of patients had no previous history of cancer. Six percent of cases had a history of non-communicable diseases. Nearly 70% of cases referred themselves to this hospital (Table 3).

### Nasopharyngeal, tongue and laryngeal cancer distribution by age and sex

**Figure 1.**
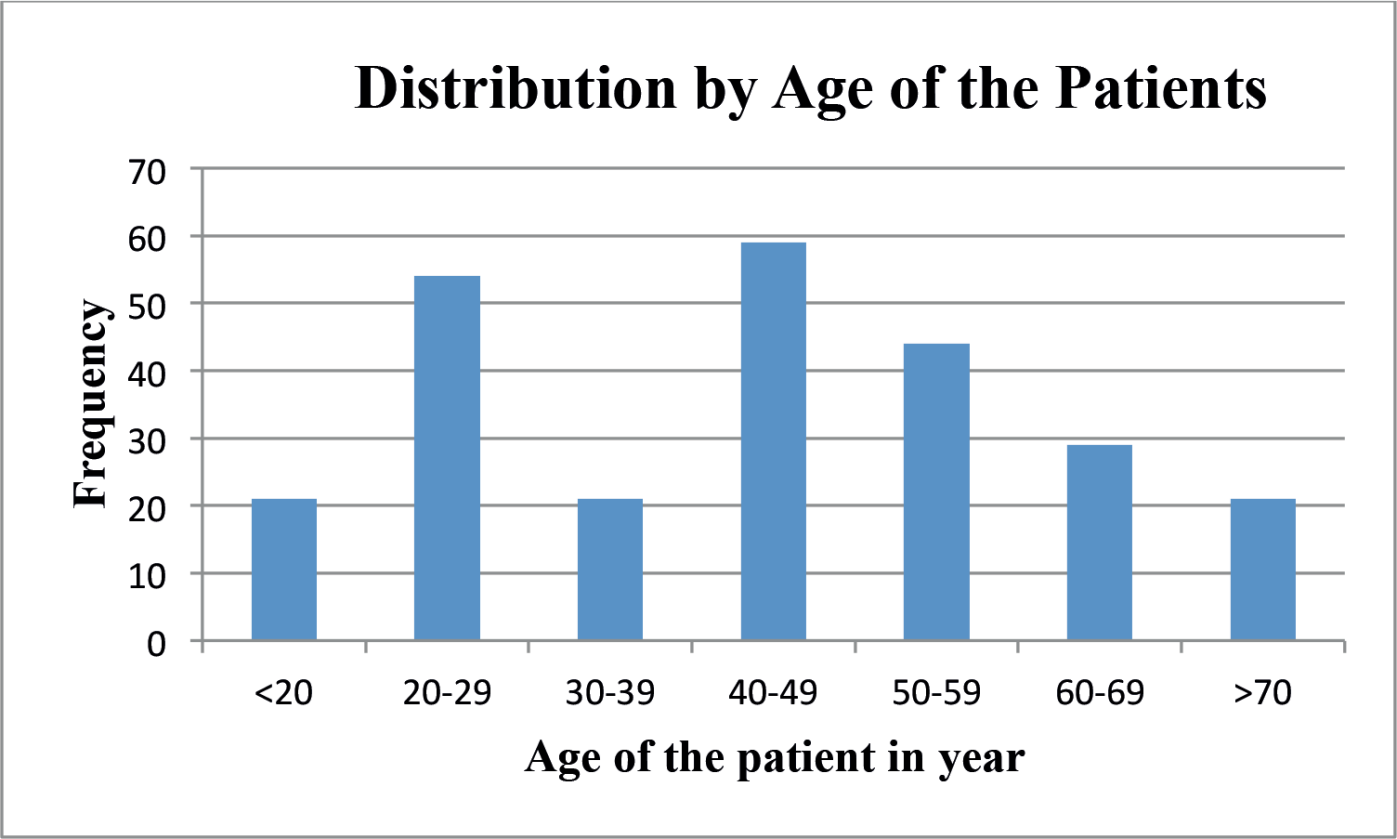
Distribution of cancer by age of nasopharyngeal, tongue, laryngeal and others HNC patients between 2013 and 2019 at HUCSH.

NPC is one of the common types of cancer categorised as HNCs and it is common in early ages less than 20 years up to 5th decades, tongue cancer was found common at the age between 3rd and 5th decades and laryngeal cancer was common at the age of 4th decades Figure 1.

Laryngeal cancer was more common among males than females with a ratio of 12.6:1 followed by nasopharyngeal of 2.2:1 and tongue of 2:1 (Table 4).

### Cancer diagnosis and characteristics

#### Nasopharyngeal cancer

The average length of time between the onset of symptoms and the NPC diagnosis was 14 months. The majority of patients with NPC (48, 64.9%) were diagnosed with fine needle aspiration cytology (FNAC) from neck lymphadenopathy. Squamous cell carcinoma was the most prevalent histologic type, accounting for 30 (62.5%) of all FNAC diagnosed cases. Of this, 63 or 85.1% of the patients had advanced cancer (stage III and IV) (Table 5).

#### Tongue cancer

An average of 17 months passed between the onset of symptoms and the tongue cancer diagnosis. The majority of tongue cancer patients [31] were diagnosed using FNAC (73.8%) from tongue lesions. Squamous cell carcinoma was the most common histologic type, accounting for 28 (90.3%) of all FNAC diagnosed cases. Of this 28 patients, or 66.7%, of the total patients, had advanced cancer (Stage III and IV) (Table 6).

The average time between the onset of symptoms and the laryngeal cancer diagnosis was 15 months. The majority of laryngeal cancer cases [19] (46.3%) were identified clinically by computerised tomography (CT) scan. Squamous cell carcinoma was the most common histologic type, accounting for 16 (94.1%) of all FNAC diagnosed cases. More than half of the laryngeal cancer patients (22 or 53.7%) had advanced cancer (Stage III and IV), and more than a quarter of the cases (11 or 26.8%) had stage II (Table 7).

#### Treatment

Because radiotherapy is not offered at HUCSH, early-stage NPC cases (stage I and II) were referred to Tikur Anbessa Specialised Hospital (TASH) for concomitant chemo radiotherapy.

Patients with locally advanced NPC (stage III) received six rounds of neoadjuvant chemotherapy consisting of cisplatin 70 mg/m^2^ IV on day 1 and gemcitabine 1,000 mg/m^2^ IV on days 1 and 8 every 21 days for six cycles. These patients were then referred for radiotherapy to TASH for radiotherapy. Stage IVNPC patients were treated with systemic chemotherapy, cisplatin 70 mg/m^2^ IV day 1 and Gemcitabine 1,000 mg/m^2^ IV day 1 and day 8 every 21 days for six cycles.

Likewise, patients with stage I and II early stage tongue and laryngeal cancer were referred to TASH for concurrent chemoradiotherapy. Patients with locally advanced tongue and laryngeal cancer (stage III) received six cycles of neoadjuvant chemotherapy consisting of cisplatin 70 mg/m^2^ IV day 1 and paclitaxel 175 mg/m^2^ IV day 1 every 21 days, followed by a referral to TASH for radiotherapy. Patients with stage IV tongue and laryngeal cancer received systemic chemotherapy, consisting of six cycles of cisplatin 70 mg/m^2^ IV day 1 and paclitaxel 175 mg/m^2^ IV day 1 every 21 days.

### Trends of nasopharyngeal, tongue and laryngeal cancers from 2013 to 2019

According to this study, the number of nasopharyngeal, tongue and laryngeal cancer cases increased from 2013 to 2019 (2.9%–32.5%). In addition, the study found that the number of cases of nasopharyngeal, tongue and laryngeal cancer had increased more than a fold since 2016. For instance, 27 (9.5%) in 2016, 67 (23.9%) in 2017 and 91 (32.5%) in 2019 Figure 2.

## Discussion

The case of nasopharyngeal, tongue and laryngeal cancer was steadily increasing from year to year, with a lot of variation (2.9%–32.5%). It is almost identical to research conducted at TASH in Addis Ababa (9.72%) [31] and in Africa [32]. The similarities in living habits and personal living standards of the study population in both Tikur Anbesa and all the African communities are probable reasons for the consistency of the findings.

NPC accounts for more than one-quarter of all HNCs (26.4%), followed by tongue (15%) and laryngeal (14.6%) malignancies. The findings of a study done in TASH were similarly congruent with this study, with a nasopharyngeal prevalence of 27% [31]. A Pakistani study conducted on the Epidemiological Review of HNCs in Karachi indicated that oral cancer cases accounted for 30% of all HNCs, followed by nasopharyngeal and laryngeal cancers in those under the age of 40 years [23] which is consistent with the recent study However, according to a study conducted in Nigeria, NPC accounts for only 12% of all HNC cases [33]. Further explanation of this difference might need a large setting study.

NPC (2.2:1), which was found common in both populations in earlier studies [34], Similarly, other studies found that the male-to-female ratio for NPC was the same (2.3–2.5:1) [3, 26]. The large number of cases and the short time of the evaluation resulted in a wide range of findings.

The majority of nasopharyngeal, tongue and laryngeal cancers were found in adults aged 20 to 40 in this study. While nasopharyngeal and tongue cancers were more likely among people over the age of twenty. NPC is more likely in those under 30 who are active and productive [21, 26].

The most prevalent cancer histology of nasopharyngeal, tongue and laryngeal cancer in this study was squamous cell carcinoma. This is in line with research conducted in Nigeria and Niger [27, 33].

Most nasopharyngeal, tongue and laryngeal cancer patients visited the hospital once their diseases had progressed to advanced stage. This outcome is consistent with research from Nigeria and South Africa [10, 11]. This may be due to the nature of the disease, which causes no discomfort until it has progressed, or it may be because the community does not have a regular annual or biannual medical exam routine or it may be a lack of cancer care services and community awareness about these cancers.

The number of nasopharyngeal, tongue and laryngeal cancer cases was rising year over year. Perhaps further research is required to determine the root cause and contributing elements. The majority of studies agreed with this conclusion [11, 14, 24, 32].

The rising rate of tongue, laryngeal and NPCs underscores serious public health issues, especially in areas like Oromia and SNNPR. For many individuals, the advanced stages of presentation and delayed diagnosis highlight the need for enhanced screening initiatives and increased public awareness. Research has demonstrated that treatment outcomes and survival rates are much enhanced by early diagnosis [35]. Furthermore, although its efficacy may vary between serum and saliva samples, the discovery of biomarkers such as Epstein-Barr virus nuclear antigen-1 (EBNA1) in patients with NPC offers interesting avenues for monitoring therapy efficacy [36].

This disparity implies that even while EBNA1 might be a useful tool in clinical settings, more investigation is required to maximise its use and comprehend the underlying mechanisms influencing its presence in various body fluids. Furthermore, the correlation between HNCs and alcohol, tobacco use and HPV suggests that public health interventions aimed at these risk factors may play a critical role in lowering incidence rates [37, 38]. Therefore, the management and prognosis of head and neck malignancies may be greatly impacted by comprehensive methods that integrate biomarker utilisation, early detection and preventive interventions.

Due to a shortage of data, we were unable to report on the effectiveness of the treatment and illness progression in this study. This study also lacks genetic test results as a result of a lack of genetic test results.

## Conclusion

The study found that the burden of morbidity related to nasopharyngeal, tongue and laryngeal cancer is increasing. The distributions of nasopharyngeal, tongue and laryngeal cancer were frighteningly surging in both sexes and throughout all age categories, increasing from year to year by place, person and time. Therefore, quick reactions are crucial everywhere to track the disease’s rapid spread. A crucial step in monitoring progress is the establishment of a population-based and institutional cancer registry, and expanding cancer centers and awareness creation activities throughout the country.

## List of abbreviations

AJCC, American Joint Committee on Cancer; FNAC, Fine needle aspiration cytology; HNC, Head and neck cancer; HPV, Human papilloma virus; HUCSH, Hawassa University Comprehensive Specialised Hospital; SNNPR, Southern Nation Nationalities People and Regional State; SPSS, Statistical Package for the Social Science; WHO, World Health Organisation.

## Conflicts of interests

The authors declare that they have no competing interests.

## Funding

The authors received no specific funding for this study.

## Consent to publish

Not applicable.

## Ethical consideration

Approval of this study was given by the research and the ethics committee of the School of Public Health and the Institutional Review Board of the College of Medical and Health Sciences at Hawassa University with an official letter of the reference number IRB/027/11. Permission to undertake this study was also obtained from the Hawassa University comprehensive specialised referral hospital administrative director then an official letter was sent to the medical record department from the chief clinical service officer of the hospital. Since then, all data has been derived from secondary sources, such as medical records, with no direct interaction with patients. To protect the privacy and confidentiality of sensitive information, each participant’s name was replaced with a code.

## Availability of data and materials

The datasets used/or analysed during the current study is available from the corresponding author upon reasonable request.

## Author contributions

AG, NB, DG, NM and DD participated in planning the study, writing a proposal, monitoring the data collection process and analysing the data, and writing the result and the manuscript. All authors agreed to be accountable for all aspects of the work. All authors read and approved the final manuscript.

## Figures and Tables

**Figure 2.**
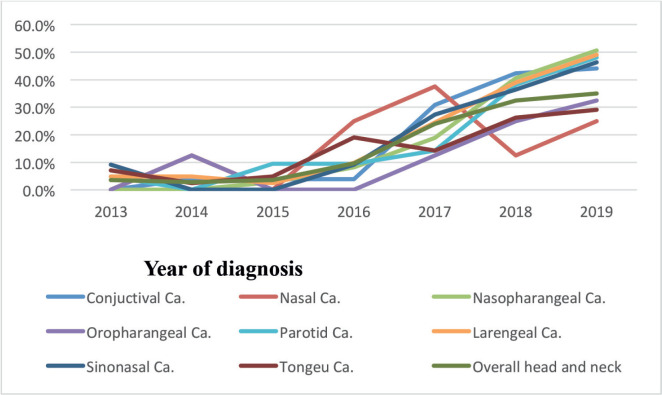
Trends of nasopharyngeal, tongue, laryngeal and others cancers patients at HUCSH from 2013 to 2019.

**Table 1.** Nasopharyngeal, tongue, laryngeal and others HNC patients distribution by body site 2013–2019 at HUCSH.

Type of HN cancer	Frequency	Percent
Naso-pharyngeal	74	26.4
Tongue	42	15
Laryngeal	41	14.6
Conjunctival	26	9.3
Parotid	21	7.5
Sino nasal	11	3.9
Nasal	8	2.9
Oropharyngeal	8	2.9
Hypo-pharyngeal	6	2.1
Buccal	6	2.1
Auricular	5	1.8
Orbital	4	1.4
Retinoblastoma	4	1.4
Tonsillar	4	1.4
Hard pallet	3	1.1
Maxillary	3	1.1
Others	14	5.1
Total	280	100

*others include – Gingival^2^, Lip^2^, mandibular^2^, oral cavity^2^, paraganglioma^1^, paranasal^2^, Soft pallet^1^ and submandibular^2^

**Table 2.** Socio-demographic characteristics of nasopharyngeal, tongue, laryngeal and others HNC patients at HUCSH between 2013 and 2019. *N* = 280.

Variable	Response	Frequency	Percent
Sex	Male	192	68.6
	Female	88	31.4
Age categorised	<30	73	26.1
	30–49	109	38.9
	50–69	79	18.2
	≥70	19	6.8
Marital status	Married	239	85.3
	Single	40	14.3
	Widowed	1	0.4
Address or region	SNNPR	157	56.1
	Oromia	121	43.2
	Somali	2	0.7

**Table 3.** Personal and previous medical history of the nasopharyngeal, tongue and laryngeal cancer patients at HUCSH between 2013 and 2019. *N* = 280.

Variable	Response	Frequency	Percent
Previous cancer history	Yes	1	0.4
	No	279	99.6
NCD	Yes	17	6.1
	No	263	93.9
Types of NCD (*N* = 17)	Diabetic mellitus	3	17.6
	Hypertension	8	47.1
	HIV	5	29.4
	Other (asthma)	1	5.9
History of tobacco smoking	Yes	4	1.4
	No	276	98.6
History of anemia	Yes	24	8.6
	No	256	91.4
Mode of referral	Self- referred	195	69.6
	Health facility referred	85	30.4

**Table 4.** Male and female ratios of nasopharyngeal, tongue, laryngeal and other HNCs between 2013 and 2019 at HUCSH.

Types of head and neck Ca.	Male	Percent	Female	Percent	M:F ratio	Total
Nasopharyngeal	51	69	23	31	2.2:1	74 (26.4%)
Tongue	28	66.7	14	33.3	2:1	42 (15 %)
Laryngeal	38	92.7	3	7.3	12.6:1	41 (14.6%)
Conjunctiva	17	65.4	9	34.6	1.9:1	26 (9.3%)
Parotid	10	47.6	11	52.4	0.9:1	21 (7.5%)
Sino nasal	6	54,5	5	45.5	1.2:1	11 (3.9%)
Nasal	3	37.5	5	62.5	0.6:1	8 (2.8%)
Oropharyngeal	5	62.5	3	37.5	1.7:1	8 (2.8%)
Others	34	69.4	15	30.6	2.5:1	49 (17.5%)
Total	192	68.6	88	31.4	2.2:1	280 (100%)

**Table 5.** Characteristics, stage and histological of NPC among study participants *N* = 74.

Variable		Frequency	Percent
Duration of symptoms	≤ 1year	23	31.1
	13 months-2 years	37	50
	>2 years	14	18.9
Means of diagnosis	FNAC	48	64.9
	Not stated	5	6.7
	Clinical diagnosis	21	28.4
Histologic types from FNAC	Squamous cell carcinoma	30	62.5
	Undifferentiated carcinoma	18	37.5
Clinical stage	Stage I	1	1.3
	Stage II	5	6.8
	Stage III	35	47.3
	Stage IV	28	
	Not stated	5	6.8

**Table 6.** Characteristics, stage and histological of tongue cancer among study participants *N* = 42.

Variable		Frequency	Percent
Duration of symptoms	≤ 1year	11	26.2
	13 months-2 years	22	52.4
	>2 years	9	21.4
Means of diagnosis	FNAC	31	73.8
	Clinical diagnosis	8	19.1
	Not stated	3	7.1
Histologic types from FNAC (N = 31)	Squamous cell carcinoma	28	90.3
	Not stated	3	9.7
Clinical stage	Stage I	4	9.5
	Stage II	7	16.7
	Stage III	17	40.5
	Stage IV	11	26.2
	Not stated	3	7.1
Tumour behaviour (N = 28)	Well-differentiated	6	21.4
	Moderately- Differentiated	10	35.7
	Poorly-differentiated	9	32.2
	Not stated	3	10.7

**Table 7.** Characteristics, stage and histological of laryngeal cancer among study participants *N* = 41.

Variable		Frequency	Percent
Duration of symptoms	≤ 1year	14	34.2
	13 months-2 years	21	51.2
	>2 years	6	14.6
Means of diagnosis	Clinical diagnosis	19	46.3
	FNAC	17	41.5
	Not stated	5	12.2
Histologic types from FNAC (N = 17)	Squamous cell carcinoma	16	94.1
	Not stated	1	5.9
Clinical stage	Stage I	3	7.3
	Stage II	11	26.8
	Stage III	14	34.2
	Stage IV	8	19.5
	Not stated	5	12.2
Tumour behavior (N = 17)	Well-differentiated	3	17.6
	Moderately- Differentiated	8	47.1
	Poorly-differentiated	5	29.4
	Not stated	1	5.9
